# A single dose ChAdOx1 nCoV-19 vaccine elicits high antibody responses in individuals with prior SARS-CoV-2 infection comparable to that of double dose vaccinated SARS-CoV-2 infection naïve individuals

**DOI:** 10.21203/rs.3.rs-1250175/v1

**Published:** 2022-01-11

**Authors:** Tesfaye Gelanew, Andargachew Mulu, Markos Abebe, Timothy A Bates, Liya Wassie, Mekonnen Tefer, Desalegn Fentahun, Aynalem Alemu, Frehiwot Tamiru, Gebeyehu Assefa, Abebe Genetu Bayih, Fikadu G Taffesse, Adane Mihret, Alemseged Abdissa

**Affiliations:** Armauer Hansen Research Institute, P.O. Box: 1005, Jimma Road, ALERT campus, Addis Ababa, Ethiopia; Armauer Hansen Research Institute, P.O. Box: 1005, Jimma Road, ALERT campus, Addis Ababa, Ethiopia; Armauer Hansen Research Institute, P.O. Box: 1005, Jimma Road, ALERT campus, Addis Ababa, Ethiopia; Department of Molecular Microbiology & Immunology, Oregon Health & Sciences University, OR, USA; Armauer Hansen Research Institute, P.O. Box: 1005, Jimma Road, ALERT campus, Addis Ababa, Ethiopia; Armauer Hansen Research Institute, P.O. Box: 1005, Jimma Road, ALERT campus, Addis Ababa, Ethiopia; Armauer Hansen Research Institute, P.O. Box: 1005, Jimma Road, ALERT campus, Addis Ababa, Ethiopia; Armauer Hansen Research Institute, P.O. Box: 1005, Jimma Road, ALERT campus, Addis Ababa, Ethiopia; Armauer Hansen Research Institute, P.O. Box: 1005, Jimma Road, ALERT campus, Addis Ababa, Ethiopia; Armauer Hansen Research Institute, P.O. Box: 1005, Jimma Road, ALERT campus, Addis Ababa, Ethiopia; Armauer Hansen Research Institute, P.O. Box: 1005, Jimma Road, ALERT campus, Addis Ababa, Ethiopia; Department of Molecular Microbiology & Immunology, Oregon Health & Sciences University, OR, USA; Armauer Hansen Research Institute, P.O. Box: 1005, Jimma Road, ALERT campus, Addis Ababa, Ethiopia; Armauer Hansen Research Institute, P.O. Box: 1005, Jimma Road, ALERT campus, Addis Ababa, Ethiopia

**Keywords:** ChAdOx1 nCoV-19, SARS-CoV-2, vaccine, dose, RBD, naïve, prior infection

## Abstract

**Background:**

A single dose COVID-19 vaccines, mostly mRNA-based vaccines, are shown to induce robust antibody responses in individuals who were previously infected with SARS-CoV-2, suggesting the sufficiency of a single dose to those individuals. However, these important data are limited to developed nations and lacking in resource-limited countries, like Ethiopia.

**Methods:**

We compared receptor-binding domain (RBD)-specific IgG antibodies in 40 SARS-CoV-2 naïve participants and 25 participants previously infected with SARS-CoV-2, who received two doses of ChAdOx1 nCoV-19 vaccine. We measured the antibody response in post-vaccination blood samples from both groups of participants collected at four different post-vaccination time points: 8- and 12-weeks after each dose of the vaccine administration using an in-house developed ELISA.

**Results:**

We observed a high level of anti-RBD IgG antibodies titers 8-weeks after a single dose administration (16/27; 59.3%) among naïve participants, albeit dropped significantly (p<0.05) two months later, suggesting the protective immunity elicited by the first dose ChAdOx1 nCoV-19 vaccine will likely last for a minimum of three months. However, as expected, a significant (p<0.001) increase in the level of anti-RBD IgG antibodies titers was observed after the second dose administration in all naïve participants. By contrast, the ChAdOx1 nCoV-19 vaccine-induced anti-RBD IgG antibody titers produced by the P.I participants at 8- to 12-weeks post-single dose vaccination were found to be similar to the antibody titers seen after a two-dose vaccination course among infection-naïve participants and showed no significant (p>0.05) increment following the second dose administration.

**Conclusion:**

Taken together, our findings show that a single ChAdOx1 nCoV-19 dose in previously SARS-CoV-2 infected individuals elicits similar antibody responses to that of double dose vaccinated naïve individuals. Age and sex were not associated with the level of vaccine-elicited immune responses in both individuals with and without prior SARS-CoV-2 infection. Further studies are required to assess the need for a booster dose to extend the duration and amplitude of the specific protective immune response in Ethiopia settings, especially following the Omicron pandemic.

## 1. Background

COVID-19 continues to be a major public health concern, causing severe illness and deaths in Ethiopia and the rest of the world alike. Mass vaccination against SARS-CoV-2 is the most effective public health intervention to protect against morbidity and mortality related to SARS-CoV-2 infection [1]. Safe, efficacious, and licensed COVID-19 vaccines, including ChAdOx1 nCoV-19 (AZD1222; Oxford–AstraZeneca) are available [2–5], despite being challenged by the recurrent emergence of new SARS-CoV-2 variants. Real-world vaccine efficacy studies from developed countries have shown that the current vaccines are able to generate effective humoral and cellular immunity, albeit differential responses are observed between vaccine-induced immunity and hybrid (vaccine-induced immunity combined with natural infection) immunity [6, 7]. Several correlates of protection studies have demonstrated that higher antibody titers are associated with decreased risk of subsequent symptomatic SARS-CoV-2 infection [8–12], and several studies from developed countries have revealed the rapid waning of antibody levels among SARS-COV-2 infection naïve vaccine recipients compared to those individuals with hybrid immunity [7, 13–16]. Despite the importance of immune durability data for guiding national vaccination strategies, there is a dearth of studies from Ethiopia and other African countries looking at more locally relevant populations.

The ChAdOx1 nCoV-19 vaccine utilizes a replication-deficient adenoviral vector that induces expression of SARS-CoV-2 spike (S) protein in host cells, particularly in the skeletal muscle [17]. Vaccinated individuals subsequently generate antibodies against the spike protein, including those that target the receptor-binding domain (RBD), which contains many neutralizing epitopes. However, they do not generate antibodies against other SARS-CoV-2 structural and non-structural proteins, such as nucleocapsid (N) [18]. Studies have shown a strong correlation between anti-RBD IgG titers and SARS-CoV-2 neutralizing titers [15]. Therefore, in resource-limited countries, it is advantageous to use anti-RBD IgG testing as a proxy for virus neutralization to assess the protection offered by the ChAdOx1 nCoV-19 vaccine.

As part of the strategy to evaluate the Ethiopian national COVID-19 response through vaccination, we established a longitudinal cohort of healthcare professionals working at the Armauer Hansen Research Institute (AHRI), with and without evidence of prior SARS-CoV-2 infection and determined their levels of ChAdOx1 nCoV-19 vaccine-induced anti-RBD IgG titers across four-time points. The present study generated evidence of the duration of ChAdOx1 nCoV-19 vaccine-induced humoral responses and the long-term effect of prior SARS-CoV-2 infection on subsequent vaccine-induced responses.

## 2. Methods

### 2.1 Study Design and Participants

**Figure 1:**
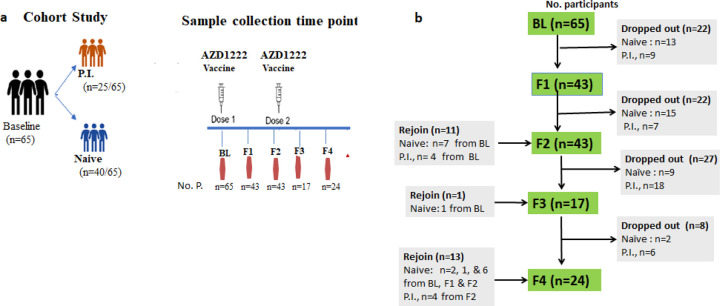
Study design with the timeline for vaccination and sample collection. (b) Schematic representation of number participants at baseline and at four postvaccination time points. Light red color character **(Naïve)** represents participants with SARS-CoV-2immunologically naïve and blue color (**P.I.**) represents participants with likely previous SARS-CoV-2 infection; **BL** =baseline or prevaccination; **F1**= 8-weeks after the first dose; **F2**=12-weeks after the first dose; **F3** =8-weeks after the second dose; and F4 =8-weeks after the second dose. The first dose and second dose were given to participants at BL and F3, respectively prior to blood sample collection.

We conducted a longitudinal prospective study constituting healthcare professionals from AHRI, who were also one of the priority target recipients of the ChAdOx1 nCoV-19 vaccine. Vaccination was offered through the Ethiopian Ministry of Health national COVID-19 vaccination campaign. In this analysis, only participants who were vaccinated with the ChAdOx1 nCoV-19 vaccine were included. The study protocol was reviewed and approved by the AHRI/ALERT Ethics Review Committee (PO/32/20) and only voluntary participants and who were able to give written informed consent were included in the study. About 5ml of venous blood was collected from a total of 65 (n=26 female and n=39 male) participants, aged 24–59 years (mean of 38.1 ± standard deviation of 8.36) before vaccination and four subsequent post-vaccination follow-ups (Figure 1) to monitor humoral responses to the vaccine. The first (F1) and second follow-ups (F2**)** were conducted at 8-and 12-weeks after the first dose, respectively, whereas the third (F3) and fourth (F4) follow-ups were done at 4 and 8 weeks after the second dose, respectively (Figure 1). All participants received their first dose between March 23, 2021 and March 31, 2021. The interval between the first and the second doses was 12 weeks (range 83–97 days). Participants were then stratified into two groups based on previous exposure to SARS-CoV-2 before vaccination and hereafter denoted as naïve and P.I. (Figure 1). The two groups: naïve (n=40) and P.I. (n=25) contained similar distributions of age and sex (Figure1 and Table 1). Table 1 summarizes the demographics of each group of study participants. In addition, participants completed a questionnaire at each visit regarding their history of RT-PCR confirmed SARS-CoV-2 infection.

### 2.2 ELISA Methods:

Prior to analyzing the presence of anti-RBD IgG antibodies, each serum sample was treated with Triton X-100 at the final concentrations of 1.0% and incubated at room temperature (RT) for 30 minutes. This procedure was performed aiming to reduce risk from any potential virus in serum [19]. Detection of anti-RBD IgG antibodies in the sera was done using a validated in-house ELISA as described previously [20]. To determine the end titer in seropositive serum samples, a two-fold serial dilution starting at 1: 200 in a 96-well ELISA plate was done. The end titer was defined as a serum dilution at which the observed optical density (OD) at 450 nm reads matched to the OD_450_ readout for pre- COVID-19 serum sample diluted at 1:200 and included in each ELISA run as a negative control. Inter-assay variability was normalized by including a convalescent serum in each run of titration ELISA.

Since the magnitude and durability of SARS-CoV-2 antibody response has been shown to positively correlate with the severity level of COVID-19 [14, 21] and yet we did not have clinical records of our P. I. participants. Thus, a convalescent sera panel (CSP; n=15) was collected from COVID-19 recovered individuals, three-months after being discharged from the Saint Paulo’s Millennium College Hospital in 2020 (before the rollout of the vaccines) were included in the anti-RBD IgG antibodies titers analysis. Based on the level of anti-RBD IgG titers in these CSP as a reference, we inferred whether our P.I. participants have had symptomatic or asymptomatic SARS-CoV-2 infection.

### 2.3 Statistical analysis

GraphPad Prism version 8.0 for Windows (GraphPad Software, La Jolla California USA) was used for statistical analyses. We measured anti-RBD IgG end titers ranging from 1:200 to 1:51200. Graphs were plotted using log10-transformed anti-RBD IgG end titers values. Table 1 comprised the calculated geometric means (GM) titers. To compare the mean differences of anti-RBD IgG-antibodies titers across each time point of serum collection, we used the unpaired non-parametrical t-test with p <0.05 =*; p< 0.01=**; p<0.001=***; p<0.0001****). Because some participants dropped out and others joined, the number of blood samples provided after the first dose varied among participants enrolled at baseline (Figure 1 and Table 1). Participants who did not provide any post-vaccine samples were excluded from the analysis.

## 3. Results

### Post-vaccination seropositivity

**Figure 2:**
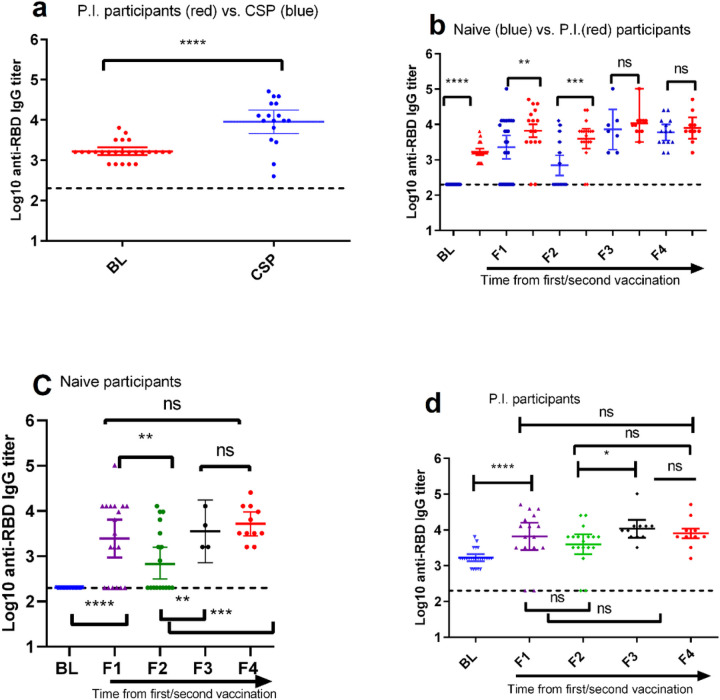
Analysis of ChAdOx1 nCoV-19 vaccine-induced antibody response in naïve and previously infected (P.I.) participants profile at different time points. Comparison of anti-RBD IgG titers profile (**a**) between P.I. participants (red dots) before they received vaccination and convalescent serum panel (CSP: blue dotes); (**b**) between naïve (blue dots) and P.I. (red dots) participants across different time points before and after vaccination; among naïve participants (**c**) and (**d**)P. I. participants across different points. **CSP**=convalescent sera panel included as a reference to indirectly assess whether P.I. participants had previously either asymptomatic or symptomatic SARS-CoV-2 infection. Each colored dot corresponds to an individual participant. Horizontal bars represent mean with 95% CI of anti-RBD IgG titer levels (transformed to Log10 value) within the indicated groups. The broken line denotes the assay detection limit. Unpaired non-parametrical t-test with p <0.05 =*; p< 0.01=**; p<0.001=***; p<0.0001=****, ns=nonsignificant) was used compare the mean differences of anti-RBD IgG-antibodies titers across each time point of serum collection. **BL**=baseline or prevaccination; **F1**= 8-weeks after the first dose; **F2**=12-weeks after the first dose; **F3** =8-weeks after the second dose; and **F4** =8-weeks after the second dose. The first dose and second dose were given to participants at BL and F3, respectively prior to blood sample collection.

Figure 1b shows the number of participants who provided a blood sample for antibody test at each post-vaccination follow-up. While detectable anti-RBD IgG antibodies 8-weeks post-first dose were measured in 16 (59.3%) naïve and 14 P.I. (87.5%) participants, we detected in 15 (60.0%) naïve and 16 (88.9%) P.I. participants at 12-weeks post-first dose (Figure 2b). At 8-weeks post-second dose, anti-RBD IgG antibodies were detected in serum samples from all the 17 (7 naïve and 10 P.I.) participants that provided blood samples. At 12-weeks post-second dose, all 24 (14 naïve and 10 P.I) participants had detectable anti-RBD IgG antibodies. Notably, four naïve (2 males with the age of 36 and 59 and 2 females aged 32 and 45 years) participants had remained seronegative at12-weeks post-first dose. On the other hand, 2 males P.I. participants aged 30 and 42 years who had higher anti-RBD IgG antibody titers at 8-weeks post-first dose underwent seroreversion at 12-weeks post-first dose.

### Comparison of anti-RBD IgG antibody titers

We first compared the pre-vaccination anti-RBD IgG titers level in P.I. participants with that of the titers in CSP. A significantly (p-value <0.0001) higher levels of anti-RBD IgG titers were observed among recovered COVID-19 patients 3-months after natural infection than in those P.I. participants (Fig. 2a).

**Figure 3:**
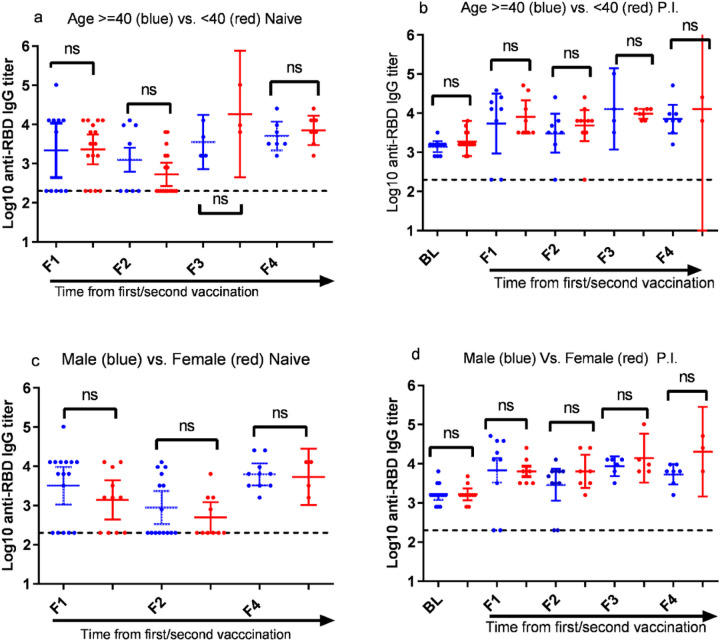
Antibody response by age and sex of naïve and previously infected (P.I.) participants following vaccination. Antibody response comparison of (a) naïve and (b) P.I. participants by age: 40–59 (blue dots) versus 21–39 years (red dots); Antibody response comparison of (c) naïve and (d) P.I. participants by sex: male (blue dots) versus female (red dots). For panel C male vs female comparison was not done at F3 since there was infection naïve female participants that provided blood sample at F3.

We also compared the levels of anti-RBD IgG titers between naïve and P.I. participants across the four post-vaccination follow-ups. P.I. had a four-fold increase at 8-weeks post-first dose compared to their pre-vaccination anti-RBD IgG antibodies levels (p=0.0004; Table 1). We also observed higher anti-RBD IgG titer levels in naïve participants at 8-weeks post-first dose, yet rapidly (30%) declined at 12-weeks post-first dose (p<0.05; Figure 2c). By contrast, high and sustainable post-first dose antibody levels were observed in P.I. participants and were found to be comparable to those naïve participants post-second dose. This trend remained consistent when results were stratified by sex and age (Figure 3).

As shown in Figure 2c, we observed a significant (p-value <0.01) increase in the level of anti-RBD IgG antibodies in naïve participants 8-weeks post-second dose compared to the level of the titer that was observed 12-weeks post-first dose. On contrary, we did not observe a statistically significant (p-value > 0.5) difference in the level of anti-RBD IgG antibodies titers in P.I. participants from those observed post-single dose (Figure 2d). Unlike the decline shown 12-weeks post-first dose, we did not see a significant (p-value >0.5) decline in mean anti-RBD IgG antibodies titers 12-weeks post-second dose in both naïve and P.I. participants (Table 1 and Figure 2b).

Figure 3 compares the levels of anti-RBD IgG antibody tiers for different age groups and sex across the four post-vaccination time points. We observed, no statistically significant difference (p-value >0.5) in the level of anti-RBD IgG titers between age 21–40 years and age 40–59 years (Figure 3a and 3b) and between males and females (Figure 3c and 3d) irrespective of participants being naïve or P.I.

## 4. Discussion

Immune protection following either vaccination or natural infection with SARS-CoV-2 decreases overtime [22]. Although the minimum antibody titer that correlates with protection has not yet been established, a decreased antibody titer is shown to be associated with an increased risk of subsequent symptomatic SARS-CoV-2 infection [8, 10–12]. In the present study, immunologically naïve participants had relatively comparable anti-SARS-CoV-2 RBD IgG responses at 8-weeks post-first dose ChAdOx1 nCoV-19 with those of participants with previous exposure to SARS-CoV-2. This finding is consistent with other studies on mRNA-based vaccines [7, 13]. Notably, at three months post-single-dose, the level of anti-RBD IgG antibodies elicited by a single dose ChAdOx1 nCoV-19 vaccine dropped significantly in individuals who were not previously infected with SARS-CoV-2. This is consistent with the suggestion made by the UK Joint Committee on Vaccination and Immunization (JCVI) -that is protective immunity elicited by the first dose ChAdOx1 nCoV-19 vaccine will likely last for a duration of 12 weeks [23]. As reported elsewhere [24], we also noted that four of our SARS-CoV-2 infection naïve participants experienced a delay in generating anti-RBD IG antibodies. Unexpectedly, we also noted seroconversion in 2 males P.I. participants aged 30 and 42 years who had higher anti-RBD IgG antibody titers 8-weeks post-first dose, indicating evidence of a rare event of rapid waning of humoral response in single-dose vaccinated individuals with prior SARS-CoV-2 infection. This could be due to critical medical conditions such as immunosuppression, though in our study, such medical conditions were not systematically recorded. Similarly, seroreversion was observed after receiving two hepatitis A vaccination in HIV–positive patients [25].

As expected, individuals who naturally contracted SARS-CoV-2 prior to vaccination developed a more rapid and sustained response to the ChAdOx1 vaccine than immunologically naïve individuals. The vaccine-induced anti-RBD IgG antibody titers produced by participants with prior SARS-CoV-2 infection at three months after a single dose vaccination appeared to be comparable to the antibody titers levels seen after a two-doses for infection-naïve participants. Similar findings have been previously reported for the ChAdOx1 nCoV-19 [26] and BNT162b2 vaccines [7, 14, 15]. Similarly, SARS-CoV-2 immunologically naïve individuals developed relatively higher and durable anti-RBB IgG antibody titers after the second dose vaccination. However, we did not observe a statistically significant difference in antibody response after second dose between participants with and without evidence of previous SARS-CoV-2 infection. Our findings, along with other studies [6, 15, 27, 28] suggest a single vaccine dose in previously infected individuals elicits similar antibody responses to that of double dose vaccination.

Participants with SARS-CoV-2 antibodies at baseline before the first vaccine injection, regardless of their sex and age (ranged 21–59), have developed strong anti-RBD IgG antibodies to the COVID-19 vaccine with no statistically significant variability between the first and the second dose. Given the age range (21–59 years with an average of 38.1 years) of our participants, this finding not surprising; however, an age-dependent decreasing pattern of anti-RBD IgG antibodies titers was reported across similar age groups to our study [29, 30]. On the other hand, the absence of statistically significant antibody titers difference between male and female participants is surprising and inconsistent with previous reports [27, 30] yet agrees with the finding reported by Wheeler et al. [31] and Lee et al.[32].

Our findings are the first evidence from Ethiopia supporting the superiority of “hybrid immunity” in eliciting a strong immunity against SARS-CoV-2 infection, comparable to that of two doses of the ChAdOx1 nCoV-19 vaccine in infection naïve individuals. Recent studies have demonstrated that sera from mRNA-vaccinated individuals with prior infection provide broader cross-neutralizing antibodies against several SARS-COV-2 variants, including Delta variants [33]. Consistent with this, another recent study revealed the mechanism by which hybrid immunity improves B cells and antibodies against SARS-CoV-2 variants[34]. However, a study revealed that the anti-spike antibody titers of BNT162b2 recipients were remarkably higher than those of ChAdOx1 nCoV-19 recipients [13]. Additionally, recent studies showed increased risk of SARS-CoV-2 Omicron infection in both vaccinated and previously infected individuals through evasion of vaccine- or infection-induced immune response and suggested the need of the rapidly developing new, Omicron variant-specific vaccine (refs). It also remains to be determined if the hybrid immunity elicited by the ChAdOx1 nCoV-19 vaccine will effectively protect vaccinees from the subsequent infections with different SARS-CoV-2 spike variants. Thus, further studies that aim to find a correlation between the level of ChAdOx1 nCoV-19 or any vaccine-induced antibody titer and clinical outcome are required.

## 5. Conclusions

Our findings demonstrate that administrating only one dose vaccine to individuals previously infected with SARS-CoV-2 infection could elicit antibody response comparbale to that of two doses of ChAdOx1 nCoV-19 vaccinated SARS-CoV-2 naïve infection and an effective strategy to stretch the supply of vaccines. Age and sex were not associated with the level of vaccine and hybrid immune responses. However, our findigs should not be translated to the Omicron variant as our study was not designed to determine the effectivenes of vaccine-elicited antibodies against Omicron. Thus further studies that compare neutralization of Omicron by sera of 1-dose vaccinated individuals with prior infection versus 2-dose vaccinated, 3-dose vaccinated naïve individuals and vaccinated individuls with Omicron breakthrough infection are required to asses the need of third booster dose over Omicron-specific booster dose in Ethiopia seetings especialy following the Omicron pandemic.

This study is limited the by number of participant dropouts, lack of neutralization assays, lack of T-cell data and by our limitation to employ N-protein-based antibody test verification for indirect evidence of breakthrough infections.

## Figures and Tables

**Table 1:** Baseline characteristics and serostatus of study participants (n = 65) and longitudinal humoral immune response

Overall (n=65)				
Character	n^a^	Mean (SD^b^)	%	GMTs^c^
Age in years		38.1 (8.4)		
<40			58.5	
>=40			41.5	
Sex				
Male	29		60.0	
Female	39		40.0	
Naïve^d^	25		61.5	
BL^e^	**39**			200.00
F1^f^	27			2267.68
F2^g^	25			699.73
F3^h^	7			7186.10
F4^i^	14			5966.97
P.I.^j^	40		38.5	
BL	25			1671.88
F1	15			6783.12
F2	18			4219.28
F3	11			7972.68
F4	10			5542.56

a n=number participants

b SD=standard deviation

c GMTs=geometric mean titers

e BL=baseline or prevaccination

f F1= 8-weeks after the first vaccination

g F2=12-weeks after the first dose

h F3 =8-weeks after the second vaccination

i F4 =8-weeks after the second vaccination

d naïve= participants without previous exposure to SARS-CoV-2 infection

j P.I.= participants with previously infected with SARS-CoV-2 infection.

## References

[1] Hasan T, Beardsley J, Marais BJ, Nguyen TA, Fox GJ. The Implementation of Mass-Vaccination against SARS-CoV-2: A Systematic Review of Existing Strategies and Guidelines. Vaccines (Basel). 2021;9:326. 33915829 10.3390/vaccines9040326 PMC8066252

[2] Frater J, Ewer KJ, Ogbe A, Pace M, Adele S, Adland E, Safety and immunogenicity of the ChAdOx1 nCoV-19 (AZD1222) vaccine against SARS-CoV-2 in HIV infection: a single-arm substudy of a phase 2/3 clinical trial. Lancet HIV. 2021;8:e474–85. 34153264 10.1016/S2352-3018(21)00103-X PMC8213361

[3] Baden LR, El Sahly HM, Essink B, Kotloff K, Frey S, Novak R, Efficacy and Safety of the mRNA-1273 SARS-CoV-2 Vaccine. N Engl J Med. 2020;:NEJMoa2035389. 10.1056/NEJMoa2035389 PMC7787219 33378609

[4] Xia S, Zhang Y, Wang Y, Wang H, Yang Y, Gao GF, Safety and immunogenicity of an inactivated SARS-CoV-2 vaccine, BBIBP-CorV: a randomised, double-blind, placebo-controlled, phase 1/2 trial. Lancet Infect Dis. 2021;21:39–51. 33069281 10.1016/S1473-3099(20)30831-8 PMC7561304

[5] Sharif N, Alzahrani KJ, Ahmed SN, Dey SK. Efficacy, Immunogenicity and Safety of COVID-19 Vaccines: A Systematic Review and Meta-Analysis. Front Immunol. 2021;12:714170. 34707602 10.3389/fimmu.2021.714170 PMC8542872

[6] Gobbi F, Buonfrate D, Moro L, Rodari P, Piubelli C, Caldrer S, Antibody Response to the BNT162b2 mRNA COVID-19 Vaccine in Subjects with Prior SARS-CoV-2 Infection. Viruses. 2021;13:422. 33807957 10.3390/v13030422 PMC8001674

[7] Angyal A, Longet S, Moore SC, Payne RP, Harding A, Tipton T, T-cell and antibody responses to first BNT162b2 vaccine dose in previously infected and SARS-CoV-2-naive UK health-care workers: a multicentre prospective cohort study. The Lancet Microbe. 2021;0. 10.1016/S2666-5247(21)00275-5 PMC8577846 34778853

[8] Addetia A, Crawford KHD, Dingens A, Zhu H, Roychoudhury P, Huang M-L, Neutralizing Antibodies Correlate with Protection from SARS-CoV-2 in Humans during a Fishery Vessel Outbreak with a High Attack Rate. J Clin Microbiol. 2020;58:e02107–20. 32826322 10.1128/JCM.02107-20 PMC7587101

[9] Huang AT, Garcia-Carreras B, Hitchings MDT, Yang B, Katzelnick LC, Rattigan SM, A systematic review of antibody mediated immunity to coronaviruses: kinetics, correlates of protection, and association with severity. Nat Commun. 2020;11:4704. 32943637 10.1038/s41467-020-18450-4 PMC7499300

[10] Rajesh T. Gandhi MD. Toward Defining an Immune Correlate of Protection Against SARS-CoV-2. NEJM Journal Watch. 2021;2021.

[11] Sui Y, Bekele Y, Berzofsky JA. Potential SARS-CoV-2 Immune Correlates of Protection in Infection and Vaccine Immunization. Pathogens. 2021;10:138. 33573221 10.3390/pathogens10020138 PMC7912691

[12] Feng S, Phillips DJ, White T, Sayal H, Aley PK, Bibi S, Correlates of protection against symptomatic and asymptomatic SARS-CoV-2 infection. Nat Med. 2021;27:2032–40. 34588689 10.1038/s41591-021-01540-1 PMC8604724

[13] Shrotri M, Navaratnam AMD, Nguyen V, Byrne T, Geismar C, Fragaszy E, Spike-antibody waning after second dose of BNT162b2 or ChAdOx1. Lancet. 2021;398:385–7. 34274038 10.1016/S0140-6736(21)01642-1 PMC8285117

[14] Levi R, Azzolini E, Pozzi C, Ubaldi L, Lagioia M, Mantovani A, One dose of SARS-CoV-2 vaccine exponentially increases antibodies in individuals who have recovered from symptomatic COVID-19. J Clin Invest. 131:e149154. 10.1172/JCI149154 PMC8203458 33956667

[15] Carbonare LD, Valenti MT, Bisoffi Z, Piubelli C, Pizzato M, Accordini S, Antibody response to BTN162b2 mRNA vaccination in naïve versus SARS-CoV-2 infected subjects with and without waning immunity. 2021.

[16] Shenoy P, Ahmed S, Paul A, Cherian S, Umesh R, Shenoy V, Hybrid immunity versus vaccine-induced immunity against SARS-CoV-2 in patients with autoimmune rheumatic diseases. The Lancet Rheumatology. 2021;0. 10.1016/S2665-9913(21)00356-8 PMC8608390 34841270

[17] Watanabe Y, Mendonça L, Allen ER, Howe A, Lee M, Allen JD, Native-like SARS-CoV-2 Spike Glycoprotein Expressed by ChAdOx1 nCoV-19/AZD1222 Vaccine. ACS Central Science. 2021;:9. 10.1021/acscentsci.1c00080 PMC8043200 34056089

[18] Yu J, Tostanoski LH, Peter L, Mercado NB, McMahan K, Mahrokhian SH, DNA vaccine protection against SARS-CoV-2 in rhesus macaques. Science. 2020;369:806–11. 32434945 10.1126/science.abc6284 PMC7243363

[19] Isho B, Abe KT, Zuo M, Jamal AJ, Rathod B, Wang JH, Persistence of serum and saliva antibody responses to SARS-CoV-2 spike antigens in COVID-19 patients. Sci Immunol. 2020;5:eabe5511. 33033173 10.1126/sciimmunol.abe5511 PMC8050884

[20] Gelanew T, Seyoum B, Mulu A, Mihret A, Abebe M, Wassie L, High Seroprevalence of Anti-SARS-CoV-2 Antibodies Among Ethiopian Healthcare Workers. Research Square. 10.21203/rs.3.rs-676935/v1. PMC8926102 35296265

[21] Chang-Monteagudo A, Ochoa-Azze R, Climent-Ruiz Y, Macías-Abraham C, Rodríguez-Noda L, Valenzuela-Silva C, A single dose of SARS-CoV-2 FINLAY-FR-1A vaccine enhances neutralization response in COVID-19 convalescents, with a very good safety profile: An open-label phase 1 clinical trial. The Lancet Regional Health – Americas. 2021;4. 10.1016/j.lana.2021.100079 PMC8442527 34541571

[22] Israel A, Shenhar Y, Green I, Merzon E, Golan-Cohen A, Schäffer AA, Large-scale study of antibody titer decay following BNT162b2 mRNA vaccine or SARS-CoV-2 infection. medRxiv. 2021;:2021.08.19.21262111. 10.3390/vaccines10010064 PMC8781423 35062724

[23] Joint Committee on Vaccination and Immunisation (JCVI) advice on third primary dose vaccination. GOV.UK. https:/www.gov.uk/government/publications/third-primary-covid-19-vaccine-dose-for-people-who-are-immunosuppressed-jcvi-advice/joint-committee-on-vaccination-and-immunisation-jcvi-advice-on-third-primary-dose-vaccination. Accessed 21 Dec 2021.

[24] Racine-Brzostek SE, Yee JK, Sukhu A, Qiu Y, Rand S, Barone PD, Rapid, robust, and sustainable antibody responses to mRNA COVID-19 vaccine in convalescent COVID-19 individuals. JCI Insight. 6:e151477. 10.1172/jci.insight.151477 PMC8564891 34499052

[25] Huang S-H, Huang C-H, Wang N-C, Chen T-C, Lee Y-T, Lin S-P, Early Seroreversion After 2 Doses of Hepatitis A Vaccination in Human Immunodeficiency Virus–Positive Patients: Incidence and Associated Factors. Hepatology. 2019;70:465–75. 30614542 10.1002/hep.30495 PMC6767446

[26] Sasikala M, Shashidhar J, Deepika G, Ravikanth V, Krishna VV, Sadhana Y, Immunological memory and neutralizing activity to a single dose of COVID-19 vaccine in previously infected individuals. Int J Infect Dis. 2021;108:183–6. 34022331 10.1016/j.ijid.2021.05.034 PMC8132551

[27] Ibarrondo FJ, Fulcher JA, Goodman-Meza D, Elliott J, Hofmann C, Hausner MA, Rapid Decay of Anti–SARS-CoV-2 Antibodies in Persons with Mild Covid-19. N Engl J Med. 2020;:NEJMc2025179. 10.1056/NEJMc2025179 PMC7397184 32706954

[28] Frieman M, Harris AD, Herati RS, Krammer F, Mantovani A, Rescigno M, SARS-CoV-2 vaccines for all but a single dose for COVID-19 survivors. EBioMedicine. 2021;68:103401. 34051441 10.1016/j.ebiom.2021.103401 PMC8149267

[29] Bates TA, Leier HC, Lyski ZL, McBride SK, Coulter FJ, Weinstein JB, Neutralization of SARS-CoV-2 variants by convalescent and BNT162b2 vaccinated serum. Nat Commun. 2021;12:5135. 34446720 10.1038/s41467-021-25479-6 PMC8390486

[30] Abu Jabal K, Ben-Amram H, Beiruti K, Batheesh Y, Sussan C, Zarka S, Impact of age, ethnicity, sex and prior infection status on immunogenicity following a single dose of the BNT162b2 mRNA COVID-19 vaccine: real-world evidence from healthcare workers, Israel, December 2020 to January 2021. Euro Surveill. 2021;26:2100096. 10.2807/1560-7917.ES.2021.26.6.2100096 PMC7879501 33573712

[31] Wheeler SE, Shurin GV, Yost M, Anderson A, Pinto L, Wells A, Differential Antibody Response to mRNA COVID-19 Vaccines in Healthy Subjects. Microbiol Spectr. 9:e00341–21. 10.1128/spectrum.00341-21 PMC8552678 34346750

[32] Lee SW, Moon J-Y, Lee S-K, Lee H, Moon S, Chung SJ, Anti-SARS-CoV-2 Spike Protein RBD Antibody Levels After Receiving a Second Dose of ChAdOx1 nCov-19 (AZD1222) Vaccine in Healthcare Workers: Lack of Association With Age, Sex, Obesity, and Adverse Reactions. Front Immunol. 2021;12:779212. 34899739 10.3389/fimmu.2021.779212 PMC8654782

[33] Leier HC, Bates TA, Lyski ZL, McBride SK, Lee DX, Coulter FJ, Previously infected vaccinees broadly neutralize SARS-CoV-2 variants. medRxiv. 2021;:2021.04.25.21256049.

[34] Andreano E, Paciello I, Piccini G, Manganaro N, Pileri P, Hyseni I, Hybrid immunity improves B cells and antibodies against SARS-CoV-2 variants. Nature. 2021;600:530–5. 34670266 10.1038/s41586-021-04117-7 PMC8674140

